# The Role of Endoscopic Sinus Surgery in Children with Cystic Fibrosis

**DOI:** 10.3390/jcm14248835

**Published:** 2025-12-13

**Authors:** Francesca Galluzzi, Werner Garavello, Gianluca Dalfino, Francesca De Bernardi, Paolo Castelnuovo, Mario Turri-Zanoni

**Affiliations:** 1 Department of Otorhinolaryngology, Fondazione IRCCS San Gerardo dei Tintori, 20900 Monza, Italy; francesca.galluzzi@irccs-sangerardo.it; 2 Department of Otorhinolaryngology, School of Medicine and Surgery, University of Milano-Bicocca, 20126 Milan, Italy; 3 Division of Otorhinolaryngology, Department of Biotechnology and Life Sciences, University of Insubria, 21100 Varese, Italy; gianluca.dalfino@gmail.com (G.D.); francescadebernardi@hotmail.com (F.D.B.); paolo.castelnuovo@me.com (P.C.); tzmario@inwind.it (M.T.-Z.); 4 Head and Neck Surgery & Forensic Dissection Research Center (HNS&FDRc), Department of Biotechnology and Life Sciences, University of Insubria, 21100 Varese, Italy

**Keywords:** cystic fibrosis, functional endoscopic sinus surgery, chronic rhinosinusitis, nasal polyps, children, pediatric

## Abstract

**Objectives:** The aim of this study was to assess the role of functional endoscopic sinus surgery (FESS) in the treatment of chronic rhinosinusitis (CRS) in children with cystic fibrosis (CF). **Methods:** We performed a comprehensive review of the literature by searching PubMed/MEDLINE. **Results:** CRS affects most children with CF. Though subjective symptoms are variable, radiological and endoscopic examination demonstrated typical objective findings. FESS is recommended for children with significant nasal symptoms that do not respond to medical treatment. At present, there are no uniform criteria for timing and extension of surgery. Primary surgery includes nasal polypectomy and correction of any bone anatomical variants that reduce ventilation of paranasal sinuses predisposing to recurrent sinusitis and complications. In case of recurrences, revision surgery supports a more expanded surgical approach. Moreover, FESS can relieve symptoms, improve patients’ quality of life, manage complications, ameliorate the delivery of medical therapy, and reduce sinonasal and lung superinfections. **Conclusions**: FESS has emerged as a safe and effective procedure for the treatment of CRS in children with CF. Since children with CF and CRS are difficult-to-treat patients, a multidisciplinary approach in tertiary-care referral centers is required.

## 1. Introduction

Cystic fibrosis (CF) is an autosomal recessive disorder caused by a genetic mutation in the cystic fibrosis transmembrane conductance regulator (CFTR) gene on chromosome 7, which encodes for a chloride ion transporter on the apical surface of epithelial cells. There are numerous known mutations of the CFTR gene, with ΔF508 being the most common. Defective chloride ion transport leads to impaired reabsorption of sodium ions and water from the luminal surface of epithelial cells, resulting in reduced water content of secretions and the formation of viscous mucus [1,2].

As a consequence, the accumulation of thickened inspissated mucus secretions causes sinonasal mucociliary clearance alterations, chronic inflammation, bacterial colonization, and infection in multiple organ systems, including the upper and lower airways. Hence, these mechanisms predispose CF children to develop chronic rhinosinusitis (CRS) and nasal polyposis (6–48%) [3,4]. Historically, surgery included open approaches such as intranasal ethmoidectomy, Caldwell–Luc procedure, medial maxillectomy, and frontal sinus trephination, until the advent of endoscopic nasal surgery [5]. Functional endoscopic sinus surgery (FESS) is recommended to restore paranasal sinus drainage pathways, create an open and accessible sinonasal cavity for topical therapy, and potentially reduce bacterial seeding of the lungs [6]. To date, it has been recommended as a safe and effective procedure for enhancing quality of life, improving the delivery of medical therapy, and reducing sinonasal and lung superinfections [7,8]. Medical therapy is the mainstay of treatment in pediatric rhinosinusitis and includes culture-directed antibiotics, systemic oral and topical nasal steroids, and nasal irrigations. In case of failure, surgical therapy is an option for management of the disease.

Interestingly, Cystic Fibrosis Transmembrane Conductance Regulator Modulator Therapy (CFTR) has recently been introduced for the treatment of CF in children.

The available evidence has shown that CF children with at least one F508del mutation treated with Elexacaftor/tezacaftor/ivacaftor (ETI) significantly improve in terms of sinonasal symptoms and related quality of life (QoL), including the emotional domain [9]. Similarly, Bech et al., studying 23 children with CF treated with ETI, reported an improvement in the sense of smell and taste along with a decrease in the frequency of CRS [10]. From this perspective, Sedaghat suggested the necessity to modulate surgical indication given the promising role of CFTR modulators for sinonasal symptoms [11,12]. In particular, he advocated for changing the paradigm of the approach to sinonasal symptoms and sinus disease in patients with CF who are on CFTR therapy.

However, it has been considered that to date CFTR may not prevent all sinonasal manifestations of CF in all patients with CF and it must be recognized that CF patients may have or develop the same primary sinonasal disorders as anyone else [11].

In this evolving scenario, this narrative review aims to describe and discuss the role of FESS for the treatment of CRS in children with CF.

## 2. Materials and Methods

We conducted a literature search in the PubMed database for articles regarding FESS for the treatment of CRS in children with CF. The search was performed from inception to 30 September 2025. A flowchart diagram illustrates the study selection process (Supplementary Figure S1). The following search terms were used: “cystic fibrosis”, “functional endoscopic sinus surgery”, “chronic rhinosinusitis”, “nasal polyps”, “treatment”, and “children”. Keywords were combined using Boolean logical operators (AND and OR). Inclusion criteria were studies that considered surgical treatment of CRS in children with CF. Exclusion criteria were non-English-language articles and studies that included participants over 18 years. Three authors (G.F., G.D., and T.Z.M.) screened the retrieved publications at three levels: title, abstract, and full text. Reference lists were examined via a manual search to identify additional eligible studies for inclusion. We identified the articles that presented the diagnostic findings and the surgical approaches and outcomes of FESS for the treatment of CRS in children with CF, and we present a narrative synthesis of the extracted data.

## 3. Results

### 3.1. Diagnostic Findings

Clinical signs and instrumental findings in children with CF and CRS are listed in Table 1. All these data may be taken into consideration by surgeons before performing FESS. And, as of now, although the evidence is preliminary, they should be integrated by the possibility of treatment with CFTR modulators [11].

#### 3.1.1. Symptoms

Symptoms in CF children with CRS are detailed in Table 1 and are often associated with ocular symptoms and other upper aerodigestive tract manifestations. The entity of sinonasal symptoms may be extremely varied, and frequently the complaint does not correlate with disease severity seen on endoscopic examination or on computed tomography [13]. Moreover, it seems that there is a high prevalence of symptomatic CRS, but that it has a low impact on quality of life [14]. The lack of complaints may be secondary to children becoming accustomed to the chronicity of the symptoms and/or the overshadowing of nasal problems by more severe symptoms, such as in lung or gastrointestinal tract symptoms [13].

Sinonasal symptoms reported by children with CF differ by age. Gysin et al. showed that adolescents most commonly reported headaches, whereas younger patients reported nasal congestion or rhinorrhea. However, headache and facial pain are difficult to quantify in younger patients and are probably underestimated [15].

Considering patients with CRS with nasal polyps, the major complaint is nasal obstruction and hyposmia, whereas the main complaint of patients with chronic sinusitis is headache or orbital pain [13]. Additionally, older patients may experience diplopia, pyomucocele, or permanent nasal bone deformity, as seen in Woakes’ syndrome [16]. Overall, the paucity of complaints in children has made it difficult to estimate the clinical severity of sinonasal disease, as well as the outcome of sinus treatments. From this perspective, it is important to obtain a detailed history and perform a physical examination to elicit the symptoms and signs of sinonasal disease [15].

#### 3.1.2. Endoscopic Findings

**Figure 1:**
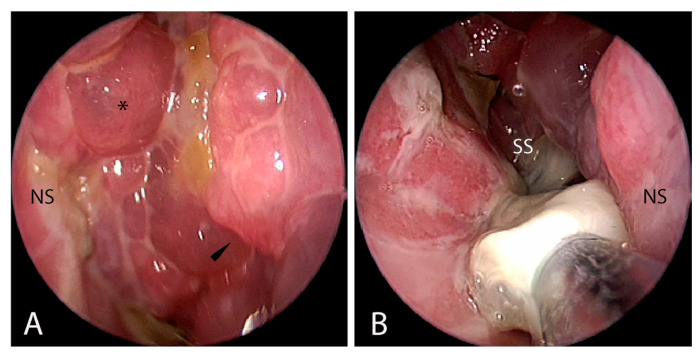
Nasal endoscopy of a 4-year-old female patient affected by CF and CRS: (**A**) in the left nasal fossa, nasal polyps and mucosal edema at the level of the left maxillary sinusotomy are visible; (**B**) in the right nasal fossa, it is possible to notice purulent and mucinous secretion at the level of the right sphenoid sinus. Abbreviations: NS, nasal septum; *, nasal polyps; black arrowhead, left maxillary sinusotomy edema; SS, sphenoid sinus. Parent/guardian consent to use clinical pictures of the patients was obtained.

Nasal endoscopy in CF children is almost always abnormal, revealing mucosal congestion, mucopurulent secretions, and nasal polyps. Table 1 details the main endoscopic findings, and Figure 1 shows a typical endoscopic view in CF children with CRS.

Nasal endoscopy is a safe procedure, well tolerated even in children [17]. It helps to obtain an endoscopic view of the extension and severity of the sinonasal inflammation, nasal obstruction, and possible superinfection. In addition, it allows the physician to perform a microbiological swab.

Robertson et al. reported that most polyps in CF are small and over 60% are not visible outside the meatus, and that endoscopy significantly improves detection of nasal polyps by 33–56.5% [13]. In children with polyposis, the bone of the uncinate process is usually destroyed by disease, leaving a floppy membrane that is ballooned medially or rotated into the nasal cavity by the polyps. Also, the middle turbinate can have a marked medialization and purulent discharge can be present [16].

Yung et al. noted that the operative findings for all children are very similar. Extensive polyps were found in the nose and almost all of the sinuses. In particular, pyocele of the ethmoid and maxillary sinuses with thick, green inspissated pus was found in all of the patients [18].

#### 3.1.3. Radiological Findings

**Figure 2:**
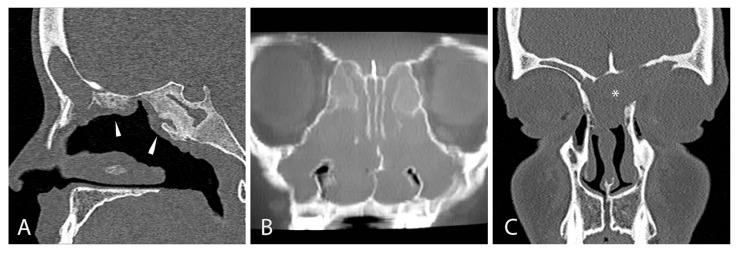
Radiological CT scan signs of chronic rhinosinusitis in 8-year-old male patients with CF: (**A**) sagittal CT scan view showing the presence of hyperostosis at the ethmoidal roof and sphenoidal sinus due to chronic inflammation, pointed out by white arrowheads; (**B**) coronal CT scan view showing bilateral massive nasal polyposis; (**C**) coronal CT scan view showing left fronto-ethmoidal empyema (*). Parent/guardian consent to use clinical pictures of the patients was obtained.

Sinus computed tomography (CT) provides excellent detail of bony and soft tissues, and several sinonasal anomalies have been described in CF children. Table 1 details the main CT findings. Typical CT scan images show opacification of the sinuses, particularly the maxillary sinus, as well as demineralization and medial displacement of the nasal wall and uncinate process, commonly resulting in mucocele [16,18]. On axial CT scan, these alterations result in the so-called “hourglass image”, which is typical of bilateral mucoceles occluding the choanae in CF patients [16]. Figure 2 shows frequently observed CT scan patterns in such patients, such as full opacification of the paranasal cavities, inflammatory hyperostosis, and bony erosion of the skull base.

In addition, hypodevelopment of the frontal and sphenoidal sinuses, without evidence of bone erosion on CT scan, is a strong radiographic indicator of CF [17].

CT scan is the imaging modality of choice to study CF children, and it is essential for guiding the endoscopic surgical procedure. Do et al. in a retrospective study including 41 CF children estimated that the modified Lund–Mackay score provides high specificity, while the Lund–Mackay score provides high sensitivity, for CF patients who require sinus surgery [19]. Based on the Lund–Mackey score, all sinus CT scans in patients with CF reveal moderate-to-severe sinus disease; therefore, it is crucial to determine when a sinus CT scan is actually necessary [20]. Gergin et al. analyzed CT scans from 832 CF children with CRS and found that a mean of 4.2 sinus scans had been performed per patient: 54% for disease evaluation and 35% for preoperative planning. Moreover, they discovered that otolaryngologists were more likely to order imaging for preoperative evaluation, and those scans were more likely to result in surgery compared with those requested by other physicians (*p* < 0.001) [20].

Although sinus CT has an invaluable role in operative planning, repetitive scanning does not appear to be a useful outcome measure for monitoring disease progression or evaluating medical or surgical treatment of CF sinus disease. McMurphy et al., studying 134 children with CF who underwent sinus CT, found that there was no significant difference between the preoperative (14.5, range 7–24) and postoperative (14.7, 8–24) Lund–MacKay score both after initial surgery (*p* = 0.99) and in subsequent scans, despite medical or surgical interventions (*p* = 0.90) [21]. Because frequent imaging in the pediatric population increases the risk of significant cumulative lifetime doses of ionizing radiation, unnecessary CT scans should be avoided. Again, McMurphy et al. suggested an otolaryngology consultation prior to imaging [21].

**Figure 3:**
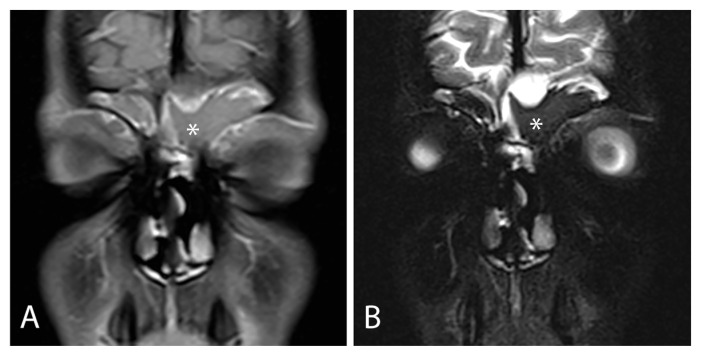
(**A**) Contrast-enhanced MRI images in T1-weighted and (**B**) T2-weighted sequences showing chronic rhinosinusitis complicated by a left fronto-ethmoidal mucocele (marked with white asterisks). Parent/guardian consent to use clinical pictures of the patients was obtained.

Magnetic resonance imaging (MRI) provides excellent soft tissue differentiation and is radiation-free. Although prior evidence suggested that MRI gave no further information in CF children with CRS [16], recent data supported MRI as a sensitive non-invasive method for diagnosis and monitoring of CRS in children with CF without radiation exposure. Sommerburg et al. performed a prospective controlled study on 67 children with CF and 30 controls underwent RM sinus with gadolinium. They found that MRI detected an increased prevalence of mucosal swelling (83% vs. 17%, *p* < 0.001), mucopyoceles (75% vs. 2%, *p* < 0.001), polyps (26% vs. 7%, *p* < 0.001), and maxillary sinus wall deformation (68% vs. 2%, *p* < 0.001) in cases compared to age-matched controls [22]. Therefore, MRI can be performed in addition to CT scan when a soft tissue complication of rhinosinusitis is suspected, as shown in Figure 3.

#### 3.1.4. Microbiological Findings

The most common bacteria isolated from the sinuses of CF patients vary with age. *Pseudomonas aeruginosa* appears to be more frequent in older patients, while *Staphylococcus aureus* and *Haemophilus influenzae* are found predominately in younger patients. Other, less common, organisms encountered are *Streptococcus* species, A. xylosoxidans, and other non-pseudomonal Gram-negative rods [23]. Anaerobes are recovered from 14.7% of CF sinus cultures, and fungi from 33.3%. Specifically, *Candida albicans* is most frequently isolated (46.2% of cases), and *Aspergillus fumigatus*, *Bipolaris* species, *Exserophilum* species, and *Penicillum* species are also found.

In CF children, various factors favor infection in the paranasal sinuses: (1) an advantageous environment in CF sinus secretions with a higher immunoglobulin (Ig)A/IgG ratio, (2) reduced inflammation, (3) low oxygen concentration, (4) bacterial biofilm formation, and (5) lower bioavailability and efficacy of intravenous antibiotic treatment compared with the lungs [17].

CRS can also contribute to the temporary or chronic lower airway infections. Indeed, a high concordance has been found between bacteria cultured from the paranasal sinuses (based on irrigations, swabs, or mucosal biopsies) and those cultured from the lungs. A concordance of 83% with regard to the presence or absence of *Pseudomonas* in the sinus and lower respiratory tract has also been reported [13,17].

A recent detailed analysis of CRS microbiota obtained from 16S rRNA gene sequencing and amplicon sequence variant (ASV) analysis reveals an unrealized diversity of CRS microbiota not captured by clinical culture. Bacterial communities dominated by *Staphylococcus* spp. were significantly more diverse compared to those dominated by *Pseudomonas* spp. CF-CRS microbiology mirrored bacterial community dynamics in the CF lung, but sinus bacterial diversity did not correlate with CRS co-morbidities [24].

### 3.2. Surgical Technique

#### 3.2.1. Goals of the Surgical Treatment

Endoscopic sinus surgery is often performed in patients with CF associated with CRS who do not respond to medical treatments and/or develop complications. The indications for surgical therapy include relieving symptoms of chronic sinusitis, reducing the frequency of exacerbations, and treating complications. Indeed, these patients typically present mucoceles or sinonasal empyemas that require surgery. Moreover, FESS allows for better access to the sinuses and therefore a better mucociliary clearance and a greater effectiveness of topical therapy with nasal rinses [25].

However, surgical therapy has an effect at the level of the sinonasal cavity and does not change the course of the underlying CF disease in any way. In addition, over the years, patients might undergo several revision surgeries due to polyps in case of CRS associated with polyps, scars, or exacerbation of CRS with complications.

Given the recent introduction of CFTR modulators for CRS in children with CF, future studies are needed to evaluate the role of surgery in these selected patients. Indeed, at present, there is no clear scientific evidence defining the timing and role of CFTR modulators in determining surgical indication for FESS in children with CF. These are relatively new therapies, and we expect that more precise indications, response criteria, and decision-making algorithms will be defined over time. It is likely that, in the future, the timing of primary FESS may be influenced by the patient’s response to CFTR modulators, potentially reducing the need for early surgery and decreasing surgical revision rates. Clinical parameters that could potentially characterize non-response to modulators include persistent nasal polyposis, thick mucopyoceles, refractory obstruction, unchanged endoscopic findings, recurrent infections, or lack of symptom/QoL improvement after 3–6 months of optimized modulator therapy. However, none of these criteria are currently universally accepted. Similarly, scenarios in which surgery may remain necessary despite modulator therapy could include complications such as mucoceles, significant anatomic obstruction, culture-positive recalcitrant infections, or children who are not eligible for ETI. This review aims precisely to open the discussion and lay the foundation for future perspectives on this evolving topic.

**Figure 4:**
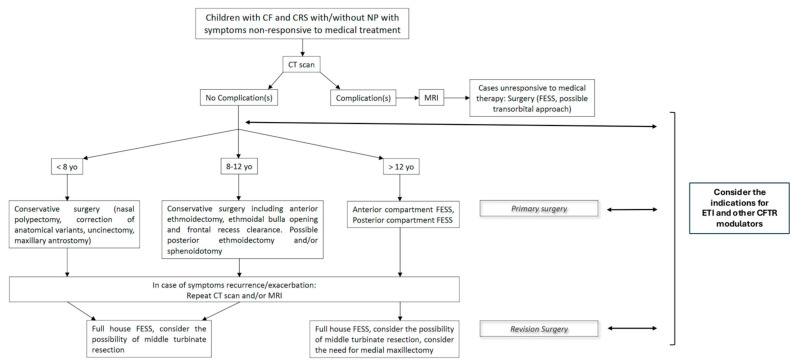
Flowchart of surgical management of CF patients with CRS. Proposed criteria for non-response to modulators are persistent nasal polyposis, thick mucopyoceles, refractory obstruction, unchanged endoscopic findings, recurrent infections, or lack of symptom/QoL improvement after 3–6 months.

Figure 4 shows the flowchart of surgical management of CF patients with CRS.

#### 3.2.2. Surgical Technique: Primary and Revision Surgery

Several studies in the literature have described the surgical management of patients with CRS associated with CF; however, there is still no standardized surgical technique, especially in the pediatric population. Table 2 reports the key studies evaluating the outcomes of FESS in children with CF. Overall, the evidence was limited. Two of the seven studies included were prospective and five retrospective. None had a control group, and the sample sizes varied among the studies.

In children, the surgical approach is almost always conservative and includes nasal polypectomy, maxillary antrostomy, and at least a minimal anterior ethmoidectomy [26]. Evidence on specific surgical techniques for the ethmoid, sphenoid, and frontal sinuses is lacking in the literature, probably because these patients often undergo subsequent revision procedures of varying extent over the years, depending on the course of the underlying mucoviscidosis.

Primary surgery includes nasal polypectomy, correction of any bone anatomical variants that may predispose recurrent sinusitis or reduce ventilation of paranasal sinuses such as Concha Bullosa, infraorbital ethmoid (Haller’s) cells, and paradoxical curvature of the middle turbinate. 

Uncinectomy, maxillary antrostomy, and irrigation of the maxillary sinus are essential, because this sinus is often occupied by dense, molded secretions due to CF.

Ventilation of the anterior sinonasal compartment should be restored by performing an anterior ethmoidectomy, opening the ethmoidal bulla, and clearing the frontal recess. When the frontal recess is occupied by thick, molded secretions that cannot be removed by sinus rinses alone, a Draf I procedure to approach the frontal sinus is useful. In cases of severe disease, it may be useful to open the second portion of the middle turbinate to make an explorative posterior ethmoidectomy to see the state of the mucous membranes and secretions in the posterior sinonasal compartment. This procedure can be used to evaluate whether to extend primary surgery with posterior ethmoidectomy and sphenoidotomy to restore ventilation of all the paranasal sinuses (full-house FESS).

There are no indications of turbinate surgery in the literature. Turbinoplasty can be performed with Argon plasma or laser in cases where the turbinate volume restricts access to the osteo-meatal complex (OMC) or to the spheno-ethmoidal recess (SER). At present, however, there is no clear consensus on this issue. Resection of the middle turbinate remains debated. Triglia et al. described resection of the middle turbinate during FESS as early as 1992 [32]. Over the years, resection of the anterior two-thirds of the middle turbinate has been described and applied several times, in association with ethmoidectomy, middle antrostomy, and, in some cases, sphenoidotomy and frontal sinusotomy [18,32].

What seems to emerge from the literature is that in primary surgery it is necessary to be as conservative as possible with regards to the middle turbinate, correcting concha bullosa or paradoxical curvature but doing everything possible to save it.

In revision surgery, instead, the recurrence of exacerbation justifies a more expanded surgical approach. In these cases, it is appropriate to perform a full-house FESS and repeated washings of all the paranasal sinuses, making sure to have created a sinusotomy with a sufficiently large caliber for the subsequent delivery of topical nasal therapy in the post-operative period. 

In this regard, some authors suggested a more aggressive approach to the maxillary sinus in recalcitrant CRS. In detail, Cho and Hwang have described the endoscopic maxillary mega-antrostomy (EMMA), a middle antrostomy enlarged posteriorly up to the posterior wall of the maxillary sinus and inferiorly up to the nasal floor, sacrificing the posterior half of the inferior turbinate [33].

Virgin et al. described a modified endoscopic medial maxillectomy (MEMM), removing the medial wall of the maxillary sinus along with the 2/3 posterior of the inferior turbinate and connecting the nasal floor with the maxillary sinus floor [25].

Shatz, finally, proposed a combined technique with an external Caldwell–Luc approach and an endoscopic one in order to obtain full access to the maxillary and ethmoid sinuses [34]. However, this latter approach is not entirely accepted in the literature due to associated morbidity.

In recalcitrant cases needing multiple revision surgeries, the reboot approach, which aims to maximally remove all sinus mucosa, has been proposed as an alternative to classical mucosa-sparing FESS for adult patients with type 2 endotype CRS with nasal polyps [35]. This extensive approach might also be theoretically applied in CF children after many revision surgeries, even if the concept of re-epithelialization of the denuded bone from the preserved healthy nasal mucosa is probably not applicable in these patients given the genetic-based mucosal pathology. At present, no case series analyzing the outcomes of reboot surgery in CF children are available in the literature. However, future studies may be helpful in this regard. It should be emphasized that the evidence supporting extended procedures such as EMMA, MEMM, the Caldwell–Luc approach, and reboot surgery in pediatric CF patients is currently limited. These techniques carry potential morbidity and should therefore be reserved for highly selected cases, ideally managed in tertiary referral centers.

Both in primary surgery and in revision surgery, the washing of the paranasal sinuses and the collection of secretions for culture examination with an antibiogram are important because, generally, there are Gram-negative multi-resistant bacteria that should be isolated and eradicated with prolonged specific antibiotic therapy in the post-operative period [30].

#### 3.2.3. Risks and Complications

FESS is considered a safe procedure which must be performed by experienced surgeons [31]. Potential complications that may occur can also be very serious and therefore should be known and avoided; these include intraoperative or postoperative hemorrhage [36]; orbital hematoma caused by bleeding from the anterior ethmoidal artery (AEA), which can be retracted into the orbit [37]; nasolacrimal duct damage during uncinectomy with subsequent transient or permanent epiphora [38]; cerebrospinal fluid leak (CSF leak) [39]; and optic nerve lesions and internal carotid artery (ICA) lesions in the sphenoid, which are very rare but theoretically possible [40].

In addition, patients may undergo more surgical revision procedures on the paranasal sinuses when they present a completely altered anatomy. In these cases, the aid of CT scanning with intraoperative navigation is essential in the surgical management of these patients in order to perform extended operations more safely and accurately. In case of revision surgery, pre-operative MRI can also be very useful, for example, when the CT scan shows a thinning of the skull base.

### 3.3. Outcomes

The results of surgery are generally evaluated, in most cases, through patient complaints, endoscopy, pulmonary function testing (PFT), need for revision surgery, and hospitalization. Most studies in the literature show a general improvement in terms of sinonasal symptoms, endoscopic appearance, and reduction in the number of hospital admissions or length of each hospitalization.

#### 3.3.1. Endoscopic/Radiological Results and Follow-Up

Children affected by CF require seriated examinations by a multidisciplinary team including a pneumologist, otorhinolaryngologist, allergist, immunologist, and pediatrician.

As for sinonasal manifestations, assessment of surgical results is mainly based on endoscopic endonasal evaluations, which should be planned more frequently in the first months after surgery (every 2 weeks or every month), and then gradually reduced to a half-yearly schedule.

In the case of infants, it can be necessary to program second-look procedures under sedation or general anesthesia [41].

During these seriated evaluations, the checklist of endoscopic parameters to be evaluated includes patency of sinusotomies, presence/absence of mucosal edema, presence/absence of nasal polyps, stagnation of nasal secretions, and type of nasal secretions (fluid or dense, infected or not).

Radiological investigations are based on massive facial CT scan without contrast, which is performed during follow-up only in selected cases when the patient’s nasal symptoms worsen or when there is a significant worsening of endonasal endoscopic parameters.

The checklist of parameters to be evaluated on CT scan includes ventilation of the paranasal sinuses, patency of the sinus ostia, recurrence of polyposis, bony thickness or erosion at the intracranial and/or intraorbital interface, and presence of hyperostosis due to inflammatory process of the bone (osteitic changes) [42].

#### 3.3.2. Association with Medical Treatment

Topical nasal medical therapy is essential and should be performed in the long term using saline nasal rinses, since the paranasal cavities are difficult to clean due to slow or absent mucociliary transport and because of the density of the nasal secretions. Both isotonic and hypertonic saline may be used, typically delivered in a high-volume and low-pressure device.

Although evidence in children with CF is scant, it also seems important to include a steroid in nasal rinses to reduce mucosal edema of the sinonasal cavities and prevent the recurrence of inflammatory polyps. The role of hyaluronic acid in nasal rinses is highly debated; it can improve mucosal healing of the sinonasal cavity and, according to some studies, improve ciliary motility. However, there is no consensus in the literature because studies are few and they do not evaluate the pediatric population [43,44].

Finally, topical antibiotic therapy can be administered through high-volume saline lavage or nebulization. This method is preferred for its capacity to reach elevated local drug concentrations within the sinonasal mucosa and biofilms while reducing systemic absorption and risks of side effects. Typical agents include tobramycin or colistin, aimed at prevalent bacteria such as *Pseudomonas aeruginosa* and *Staphylococcus aureus*. However, these patients very often have upper and/or lower airway infections and must undertake long-term systemic antibiotic therapy with intravenous antibiotics when purulent and persistent nasal secretions are present. In these cases, a culture-directed therapy is recommended; ciprofloxacin (20 mg/kg twice daily) is an oral option for P. Aeruginosa infections. Intravenous therapy is reserved for severe exacerbations or refractory disease. Long-term, low-dose macrolide therapy (e.g., azithromycin) is utilized for its immunomodulatory and anti-inflammatory properties and has shown some benefits for sinonasal symptoms and polyp size [45]

Topical nasal therapy is, obviously, complementary to medical therapy of the underlying disease, such as CFTR modulators, which also act at the sinonasal level [46]. In this regard, the promising results of CFTR modulators recently reported in children with CRS symptoms [9,10] suggest that new protocols should be developed to optimize the balance between medical and surgical treatments. At present, CFTR modulators in children have selective indications; some children may be non-responders to therapy, and others may develop sinonasal symptoms due to inflammatory responses or persistent sinonasal bacterial colonization [47].

#### 3.3.3. Impact on Quality of Life (QoL)

Several studies have shown an improvement in symptoms following FESS through standardized measures of QoL [14]. Reduced upper airway drainage and mucopurulent secretions in the lower airways and, consequently, decreased bacterial colonization of the lungs result in an improvement in sinonasal symptoms such as headaches and congestion, as well as in sleep quality. This supports the conclusion that the overall quality of life of patients with CF is improved after surgery [48]. Symptoms reported by patients or their parents are essential to assess surgical outcomes and should be scored through dedicated questionnaires, such as the Sinonasal-5 (SN-5) [49]. SN-5 is a quick and qualitative method for monitoring CRS and understanding the impact of sinus and/or nasal symptoms in children. However, the validity of the SN-5 in children with CF specifically has not been assessed in the literature. Future studies will be necessary to assess the impact of surgery in the pediatric population affected by CF through the use of validated sinonasal QoL instruments [50]. Tumin et al. pointed out that children with CF associated with CRS who undergo surgery tend to stay in the hospital longer than children without CF; however, this does not result in a higher risk of readmission to the hospital or early re-intervention [31].

Finally, there is no evidence that FESS performed in children has an effect on the outcomes of midfacial growth [51].

#### 3.3.4. Influence on Lung Function

The improvement in lung function parameters after FESS is another still debated topic. Kovell at al. reported a significant improvement in PFT scores in children with CF undergoing FESS, attributable to reduced airway obstruction, although this change may be temporary and uneven [28]. However, several studies have assessed the effect of FESS on lung function, with diverging conclusions [27]. Preoperative pulmonary infection status, FESS extension, and post-operative treatment are important parameters that need to be considered in assessing lung function. In addition, it is necessary to consider that the natural course of CF includes the progressive deterioration of lung function [29,52].

#### 3.3.5. Improvement in Nitric Oxide Level Post FESS

Finally, children affected by CRS with nasal polyps associated with CF have very low nasal nitric oxide (nNO) levels compared to those affected by CF with CRS without nasal polyps. De Winter-de Groot et al. analyzed nNO levels and lung function before and after FESS in a series of 13 children with CF, reporting a significant increase in nNO levels, which were raised not to normal levels but to levels similar to those of patients with CF without polyposis [53].

## 4. Limitations of the Current Evidence and of This Review

The existing body of evidence regarding FESS for the treatment of CRS in children with CF is constrained by several significant methodological limitations. The majority of studies are retrospective and involve small sample sizes, which inherently limits the strength of their conclusions. Furthermore, heterogeneity across the research is considerable, stemming from variations in the definition of CRS, the indications used for FESS, the extent of surgery, and the specific protocols for postoperative care. In most studies, there is a critical absence of the use of validated, pediatric CF-specific sinonasal QoL instruments, making it difficult to accurately measure patient-reported outcomes. Finally, this review has some limitations, including the exclusive use of a single database, the restriction to English-only literature, and the lack of a formal risk-of-bias assessment, all of which necessitate a cautious interpretation of the findings and highlight the need for more robust, standardized future research.

## 5. Conclusions

Children affected by CF and CRS are difficult-to-treat patients who require a multidisciplinary approach in tertiary-care referral centers. To date, FESS is a safe, repeatable, and effective surgical treatment that relieves symptoms, improves patients’ QoL, manages sinonasal infection, and potentially reduces pulmonary exacerbations. Considering the emerging role of CFTR modulators, further studies are needed to establish new surgical indications.

## Figures and Tables

**Table 1:** Diagnostic findings of CRS in children with CF.

Symptoms	Endoscopic Findings	Radiological Findings	Microbiological Findings
**Sinonasal** Nasal obstruction (81.1%) (unilateral or bilateral) Rhinorrhea (50%) Hyposmia or anosmia (27%) Headache (50%) Facial pain **Ocular** Epiphora Exophtalmia Hyperemic conjunctive **Others** Mouth breathing Chronic cough Throat clearing Halitosis Activity intolerance Agitated or restless sleep Snoring Sleep apnea Failure to thrive	Nasal mucosa congestion, particularly with turbinates Prominent uncinate process Bulging of lateral wall (unilateral or bilateral) Nasal polyps (33–56.5%) Septal deformity Choanal stenosis Viscous and/or purulent nasal secretions Adenoid hypertrophy Cobblestoning of posterior pharynx	**CT** Opacification of the sinus (hourglass image) Mucocele Bulging or displacement of lateral nasal wall Demineralization of uncinate process Hypoplasia or aplasia of the paranasal sinuses Inverse relationship between the size of anterior and posterior ethmoid sinus Decreased pneumatization of the sinus **MRI** Mucosal swelling Mucopyoceles Polyps Maxillary sinus wall deformation Delay pneumatization	**Bacteria** *Pseudomonas aeruginosa* *Staphylococcus aureus* *Haemophilus influenzae* *Streptococcus* species *Achromobacter xylosoxidans* **Fungi** *Candida albicans* *Aspergillus fumigatus* *Exserophilum* species *Penicillum* species

CT: computed tomography; MRI: magnetic resonance imaging.

**Table 2:** Key studies evaluating outcomes of FESS in children with CF.

Study (First Author, Year)	Type of Study, Setting	Sample (CF Children)	Age Range (Years)	Type/ Extent of FESS	Follow-Up	Main Outcome Measures	Main Findings	Main Limitations
Cuyler, 1992 [26]	Prospective, single center	10 CF children; 7 underwent FESS	3–19	Endoscopic sinus surgery tailored to extent of disease	2–3 years	CT appearance of sinuses; patient/parent-reported symptom change	Radiologic disease persisted or recurred in all children, but all patients/parents reported subjective symptomatic benefit	Very small sample; no control group; non-standardized symptom assessment
Rosbe, 2001 [27]	Retrospective, tertiary children’s hospital	66 children with CF (including 8 lung transplant recipients); 112 ESS procedures	mean age 17	FESS for CRS/polyposis; revision surgery in 25 patients	6–12 months	Oral/inhaled steroid use, pulmonary function tests, inpatient hospital days	No significant change in PFTs or steroid requirements; possible reduction in hospitalization days in the 6 months after surgery	Retrospective, uncontrolled; index hospitalization confounded admission data; no standardized sinonasal QoL measures
Kovell, 2011 [28]	Retrospective, tertiary CF center	62 children with CF and sinusitis; 21 underwent ESS, 41 managed medically	0–21	Extent of FESS not detailed (performed for CRS/polyposis)	Up to 3 years	FEV1% predicted, FVC% predicted; socioeconomic status	After adjustment for Medicaid status, children who underwent ESS had higher FEV1 and FVC over time compared with non-surgical patients	Non-randomized; selection bias (polyposis more common in ESS group); no direct sinonasal symptom scores
Osborn, 2011 [29]	Retrospective, single-center series	41 children with CF undergoing ESS	5–18	FESS for CRS/nasal polyps	Variable (pre- and post-operative PFTs and cultures)	Pulmonary function tests; upper and lower airway microbial cultures	ESS did not significantly improve PFTs or alter respiratory tract microbial colonization patterns	Retrospective; no control group; follow-up duration heterogeneous; quality-of-life data not collected
Do, 2014 [4]	Retrospective, tertiary pediatric hospital	153 children with CF; subset required FESS	mean age 7.1	FESS of varying extent; extent and revision rates compared across genotypes	≥1 year	Need for FESS, Lund–Mackay CT score, extent of surgery, length of stay, revision surgery	F508del genotype was not associated with increased need for FESS, greater surgical extent, or higher revision rates	Focused on genotype–phenotype; did not evaluate symptom scores or pulmonary outcomes; heterogeneous surgical techniques
Aanaes, 2013 [30]	Prospective, national CF center	106 patients with CF (children and adults)	6–50 (mixed pediatric/adult)	Extensive FESS with systematic sinus irrigation and intensive postoperative topical therapy	12 months	Sinus and lower airway bacteriology, number of positive cultures, FEV1/FVC, BMI, CF-specific antibodies, sinonasal symptoms	Significant reduction in positive lower airway cultures and intermittent colonization after FESS; only minimal changes in FEV1/FVC; sinonasal symptoms improved	Mixed adult and pediatric population (results not stratified by age); no control group; intensive adjuvant therapy makes surgical effect difficult to isolate
Tumin, 2017 [31]	Retrospective, multicenter registry study (ACS NSQIP-P)	213 children with CF vs. 821 without CF undergoing elective ESS	mean age 10	ESS as coded in registry (extent not specified)	30 days	Prolonged hospital stay (>1 day), 30-day readmission and unplanned reoperation	CF children had longer hospital stays than non-CF peers, but similar 30-day readmission and reoperation rates, supporting overall safety of ESS in CF	Registry data; no symptom, endoscopic, or radiologic outcomes; extent of surgery and CF disease severity not captured

## Data Availability

Data extracted from the included articles are available in PubMed/MEDLINE.

## Supplementary Materials

The following supporting information can be downloaded at: https://www.mdpi.com/article/10.3390/jcm14248835/s1, Figure S1: Flowchart diagram of the study selection process.

## Abbreviations

The following abbreviations are used in this manuscript:

AEA	anterior ethmoidal artery
ASV	amplicon sequence variant
CFTR	cystic fibrosis transmembrane conductance regulator
CSF	cerebrospinal fluid leak
CF	cystic fibrosis
CRS	chronic rhinosinusitis
CT	computed tomography
EMMA	endoscopic maxillary mega-antrostomy
ETI	elexacaftor/tezacaftor/ivacaftor
FESS	functional endoscopic sinus surgery
ICA	internal carotid artery
OMC	osteo-meatal complex
PFT	pulmonary function testing
MRI	magnetic resonance imaging
MEMM	modified endoscopic medial maxillectomy
nNO	nasal nitric oxide
QoL	quality of life
SN-5	sinonasal-5
SER	spheno-ethmoidal recess
