# Supplementary figures and images for: The Role of Endoscopic Sinus Surgery in Children with Cystic Fibrosis
Source: J Clin Med. 2025 Dec 13;14(24):8835. doi: 10.3390/jcm14248835 (PMC12734308; doi:10.3390/jcm14248835)

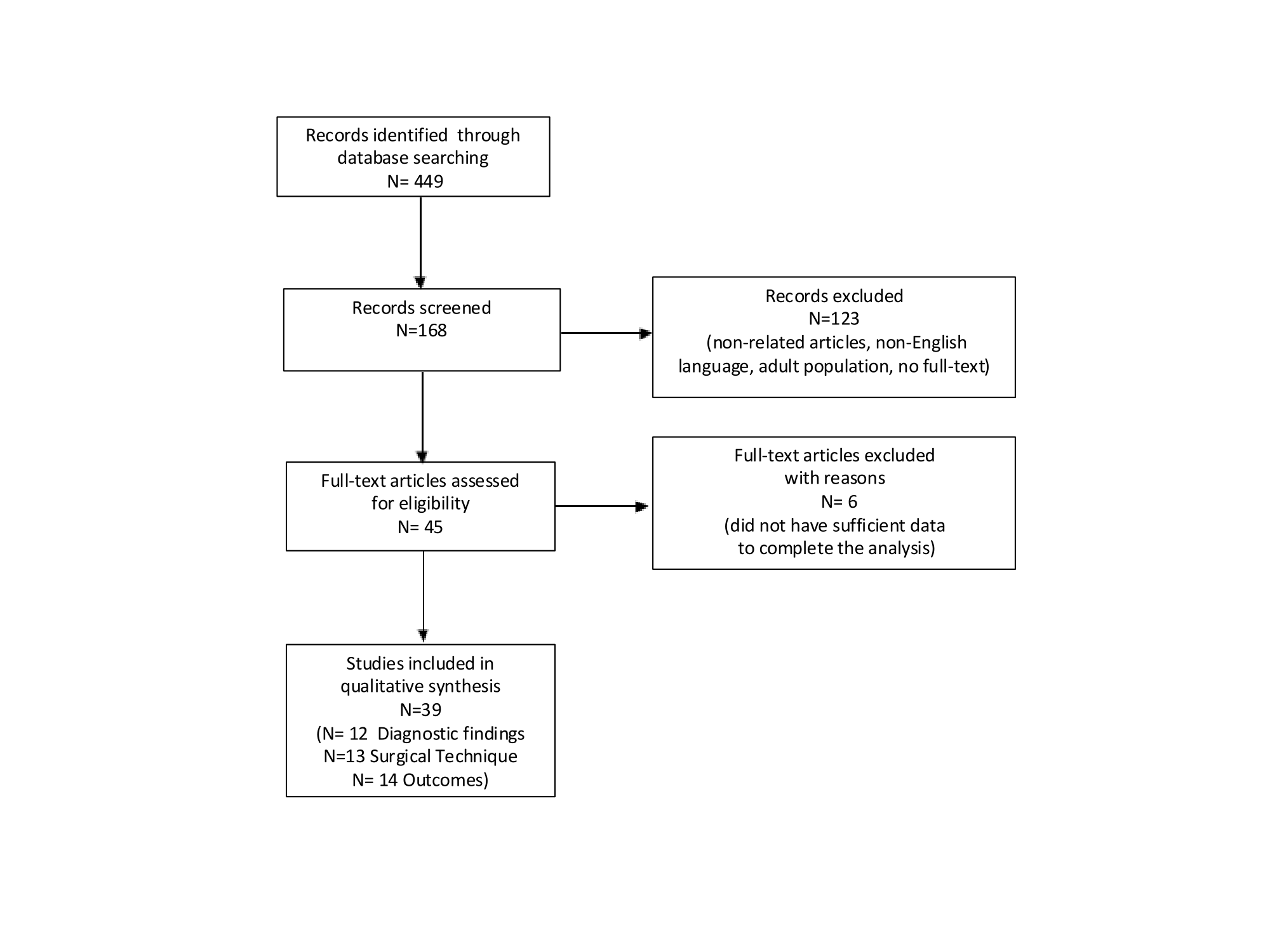

Supplement: Supplementary file 1 [file jcm-14-08835-s001.zip › jcm-3995724-supplementary.png]
